# Comparison of intravitreal bevacizumab and ranibizumab used for myopic choroidal neovascularization: A PRISMA-compliant systematic review and meta-analysis of randomized controlled trials

**DOI:** 10.1097/MD.0000000000014905

**Published:** 2019-03-22

**Authors:** Qiuming Hu, Haoyu Li, Yi Du, Jianfeng He

**Affiliations:** a Department of Ophthalmology, the First Affiliated Hospital of Medical University; b Guangxi University of Chinese Medicine, Nanning, Guangxi, China.

**Keywords:** bevacizumab, choroidal neovascularization, meta-analysis, pathologic myopia, ranibizumab

## Abstract

**Background:**

To evaluate the effect of intravitreal bevacizumab (IVB) and ranibizumab (IVR) for the treatment of choroidal neovascularization (CNV) secondary to pathologic myopia (PM) by meta-analysis.

**Methods:**

Pertinent publications of randomized controlled trials (RCTs) were identified through systemic searches of PubMed, EMBASE, Web of science, Cochrane Library, clinicaltrials.gov, CNKI, CQVIP, and Wanfang database. All comparative studies of IVB or IVR as treatment for CNV secondary to pathologic myopia were included. Meta-analysis of these RCTs was performed using Review Manager 5.3 software. The χ^2^ test and *I*^2^ values were used to analyze heterogeneity. Measurements included best-corrected visual acuity (BCVA) and central foveal thickness (CFT).

**Results:**

A total of 3 randomized controlled clinical trials involving 158 eyes were included, 81 eyes in IVB group and 77 eyes in IVR group. Compared with baseline, at 1, 3, 6, and 12 months after IVB or IVR treatment, BCVA was significantly increased. Change of BCVA at 1, 3, 6, and 12 months did not vary significantly between IVB and IVR group (1 month: *Z* = 0.30, 95% CI = −0.08 to 0.11, *P = *.76; 3 months: *Z* = 0.36, 95% CI = −0.10 to 0.15, *P = *.72; 6 months: *Z* = 0.17, 95% CI = −0.10 to 0.12, *P = *.86; 12 months: *Z* = 0.64, 95% CI = −0.15 to 0.08, *P = *.52).

**Conclusion:**

Both IVR and IVB can significantly improve BCVA of eyes with mCNV, but there was no significant difference between the 2 therapies on the treatment of mCNV.

## 1 Introduction

Macular choroidal neovascularization (CNV) formation is one of the most common complications of central vision impairment in patients with pathological myopia. The prognosis of myopic CNV (mCNV) is poor. After 5 and 10 years of onset, 89% and 96% of patients’ visual acuity reduced to 0.1 or even worse.^[1,2]^ Antivascular endothelial growth factor (VEGF) therapy has become the first-line therapy for mCNV and it might be a good treatment for nonsubfoveal CNV.^[3]^

The earliest report of intraocular injection of anti-VEGF drugs for the treatment of mCNV was in 2006 and short-term observations of this aspect become more and more common in recently years,^[4,5]^ and the conclusions support the effectiveness of anti-VEGF drugs against mCNV.^[6]^ Injection of anti-VEGF drugs has also become the first-line treatment of mCNV.^[7]^ Most previous studies have reported that intraocular injections of VEGF inhibitors have resulted in significant anatomical and functional gains in mCNV treatment, such as improved visual acuity and central dark spots, but most studies lacked randomized control settings.^[8]^

The exact difference of efficacy between bevacizumab and ranibizumab in mCNV has not been determined. To this end, we have rigorously established standards for inclusion of literature and used meta-analysis to compare the efficacy of intravitreal bevacizumab (IVB) and intravitreal ranibizumab (IVR) in improving the best corrected visual acuity (BCVA) and reducing the central foveal thickness (CFT) during treatment of mCNV.

## 2 Materials and method

### 2.1 Search strategy

Seven databases (retrieval of literature from PubMed, Embase, Web of science, the Cochrane Library, CNKI, CQVIP, and Wanfang) and clinicaltrials.gov website were last searched in November 2018. In conducing the search in the English database, retrieval keywords included pathologic myopia (myopia, degenerative), myopic choroid neovascularization, choroidal neovascularization, bevacizumab (Avastin), ranibizumab (Lucentis); “myopic choroidal neovascularization” was searched in clinicaltrials.gov. The details of search strategy were summarized in Table 1. Through the computer retrieval, the full text of the literature was obtained and further screened. Retrieved articles were imported into EndNote X8 (Thomson Reuters, New York, NY) where duplicate articles were manually removed.

**Table 1:**
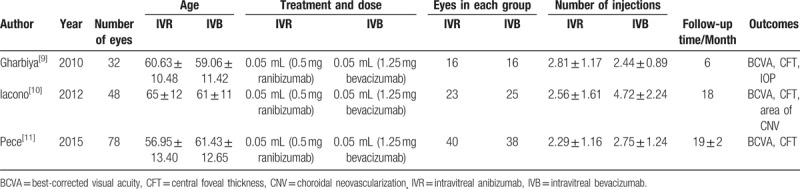
Characteristics of RCTs included.

### 2.2 Inclusion and exclusion criteria

Published studies were included if they: compared the effects between intravitreal injection of ranibizumab and bevacizumab on treatment of myopic CNV; the study sample size was not <30 eyes and reported one or more of the following outcomes at Month 1, 3, 6, 12 or longer observation time point: best-corrected visual acuity (BCVA), central foveal thickness (CFT), CNV stabilization, number of treatments, and ocular or systemic adverse events, though visual acuity was regarded as primary outcome in the subsequent data analysis; and Repetitive publications or documents of the same sample should be combined. All studies included should be randomized controlled trials and excluded potential publication bias. Studies were excluded if the subjects were not satisfied that the mCNV diagnosis or treatments were not IVB or IVR, or that no effective visits and evaluations of the treatment effects were performed; the study did not meet the RCT criteria or the grouping was unreasonable; the grouping information was incomplete.

**Figure 1:**
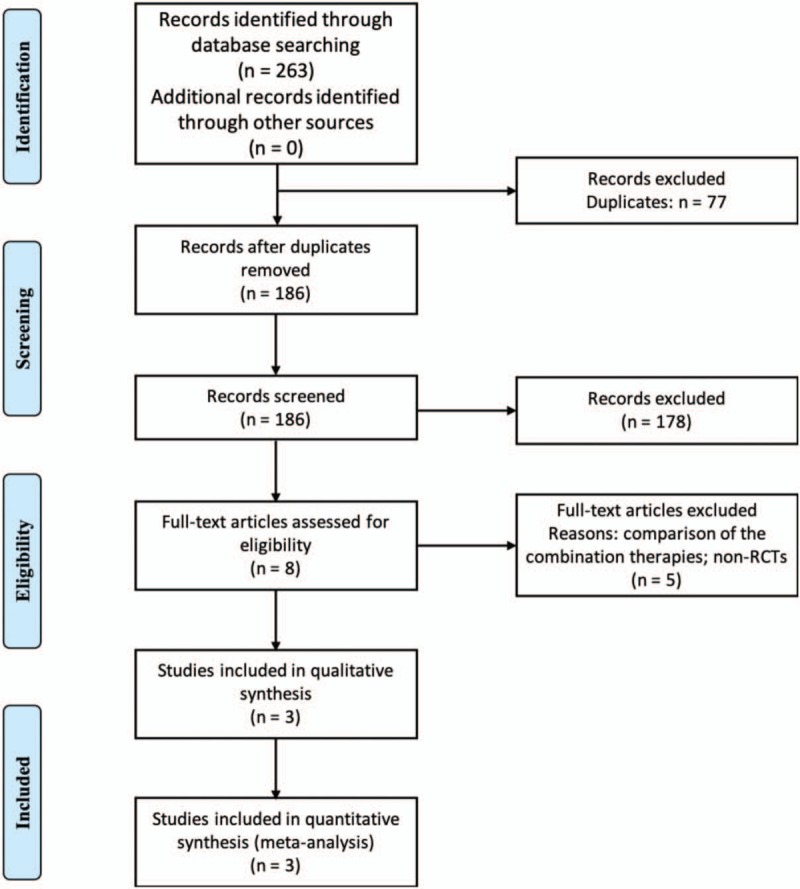
Flow diagram showing the process of study selection and categorization.

**Figure 2:**
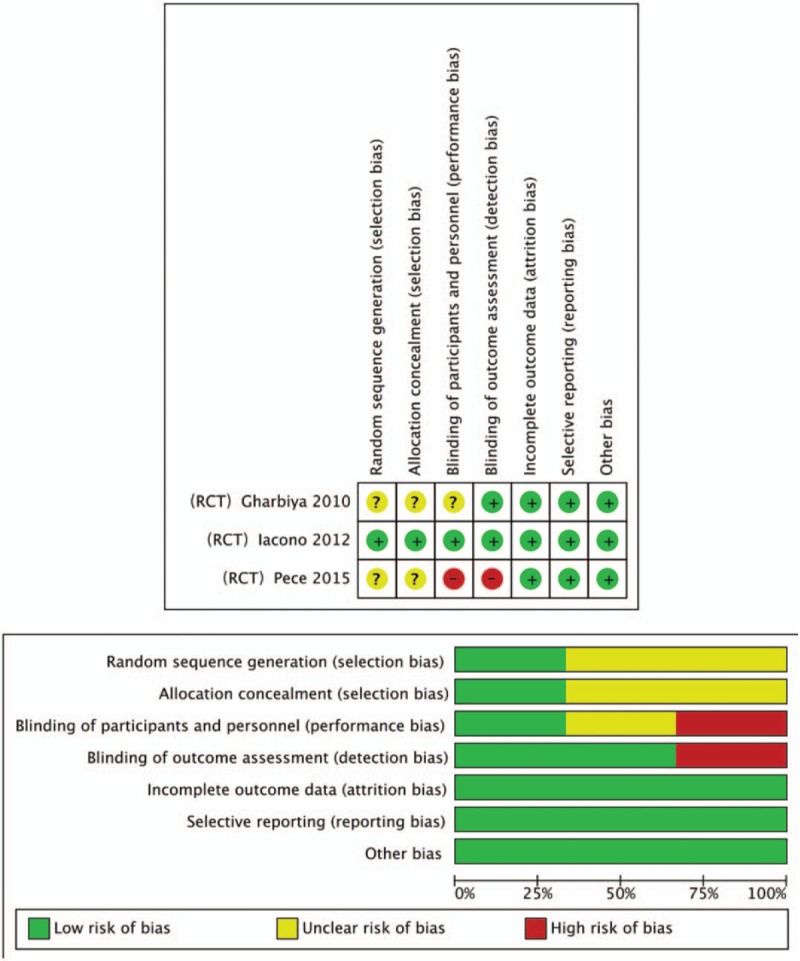
Assessment of the risk of bias in included studies. Bias risk was classified as low (+), unclear (?) or high (−).

The titles and abstracts of retrieved articles were independently scanned by 2 authors to gather information and determine whether they met inclusion criteria. Two authors resolved inconsistencies by reading full texts and consensus. A preliminary search of the target database yielded 263 articles. After removing the duplicates, 77 articles were excluded. After reading the title and abstract, 178 articles were excluded. After reading the full text, 5 articles were excluded. Finally, 3 articles that met the criteria were included in our study.^[9–11]^ The details are shown in Figure 1. The information gathered from the articles include General data: literature title, author, and time of publication; Basic characteristics of the studies: sample size, characteristics of the study subjects, treatment plans for each group and follow-up time; Outcomes: BCVA, CFT, and other outcomes. The characteristics of the 3 articles are shown in Table 2. A bias risk assessment of included studies was performed (Fig. 2).

**Table 2:**
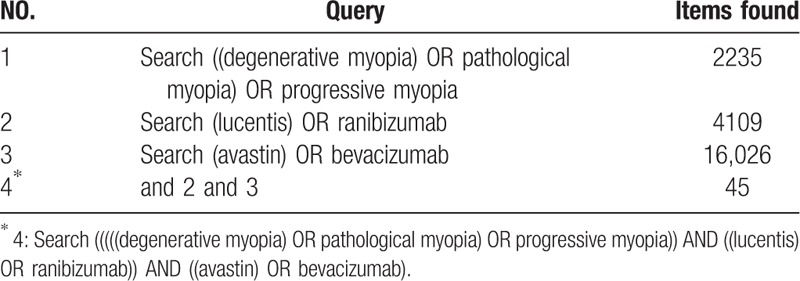
Search strategy in PubMed.

Statistical analyses were performed with Review Manager 5.3 supplied by Cochrane Collaboration (Oxford, United Kingdom). In meta-analysis, the effect sizes of each study were presented as mean difference with 95% confidence intervals (CIs) for continuous data and risk ratio (RR) with 95% CIs for discontinuous data. The meta-analysis was performed by converting the BCVA measurements to logarithmic visual acuity (logMAR). The CFT was automatically measured and calculated by the built-in software of the inspection instrument. Both BCVA and CFT are continuous variables, with a weighted mean difference (WMD) as the effect scale. The mean (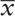) and standard deviation (s) were extracted for each group of data for combined analysis. The χ^2^ test (test level was α= 0.1) was used to test the heterogeneity of the included literature. The statistical heterogeneity was considered insignificant when *P*≥.1 of χ^2^ test or *I*^2^ statistic was ≤50%. The pooled effect sizes were considered significant when the 95% CI of weighted mean difference did not cross zero. The statistical results of the combined effects were expressed as *Z* values, and the *P* values were obtained according to the *Z* values. *P* < .05 was considered statistically significant between the 2 groups.

## 3 Results

### 3.1 Details of included studies

According to the search strategy and data collection method stated above, 263 documents were obtained from the initial inspection. Using the method of reading the title, abstract and full text of the article, documents that did not meet the inclusion criteria such as literature reviews, systematic reviews, animal experiments, letters from the readers, articles republished, single-sample uncontrolled studies or follow-up and non-RCT were excluded. Eventually, 3 eligible RCTs were included, a total of 158 eyes (79 for IVR and 79 for IVB), followed-up for 3 to 18 months. The 3 included studies are all prospective clinical randomized controlled studies. The bias assessment was performed. The performance bias is the most significant bias among the included studies. The same treatment and dose (0.05 mL 0.5 mg ranibizumab, 0.05 mL 1.25 mg bevacizumab) were used for the 3 studies. No statistically significant difference was noted regarding age, gender, BCVA, or CFT at baseline between IVR group and IVB group in 2 studies.^[9,10]^ All the baseline demographic and clinical characteristics were homogeneous between the 2 groups after adjusting the potential bias by Change Score Model in Pece's study.^[11]^

### 3.2 Efficacy evaluation of IVR and IVB

The extracted data were analyzed with Manager 5.3 software. The outcome indicators BCVA, injection times, and CFT were entered as continuous variables, CNV stabilization was entered as discontinuous variable. Two of the included studies^[9,10]^ considered that there was no significant difference in the therapeutic effect of IVR versus IVB on mCNV and one study^[11]^ was deemed necessary to increase the sample size for further study), and one study^[10]^ considered IVR to be more advantageous than IVB in the number and benefit of treatment. Besides, no major injection-related adverse events were reported in both groups in 3 included studies.

#### 3.2.1 Best corrected visual acuity (BCVA)

Among the 3 articles included, the BCVA of patients was described. A significant increase of BCVA from baseline was observed in both groups in included studies. A comprehensive analysis was performed using Review Manager 5.3 software. No heterogeneity between the IVR group and the IVB group was reported at 1 month (*P = *.23, *I*^2^ = 32%), 3 months (*P = *.36, *I*^2^ = 0), 6 months (*P = *.46, *I*^2^ = 0%) and 12 months (*P = *.87, *I*^2^ = 0%). No significant difference was reported between the 2 groups in increasing BCVA at 1 month (*Z* = 0.30, 95% CI = −0.08 to 0.11, *P = *.76), 3 months (*Z* = 0.36, 95% CI = −0.10 to 0.15, *P = *.72), 6 months (*Z *= 0.17, 95% CI = −0.10 to 0.12, *P = *.86) and 12 months (*Z *= 0.64, 95% CI = −0.15 to 0.08, *P = *.52) (Fig. 2).

#### 3.2.2 Number of injections

**Figure 3:**
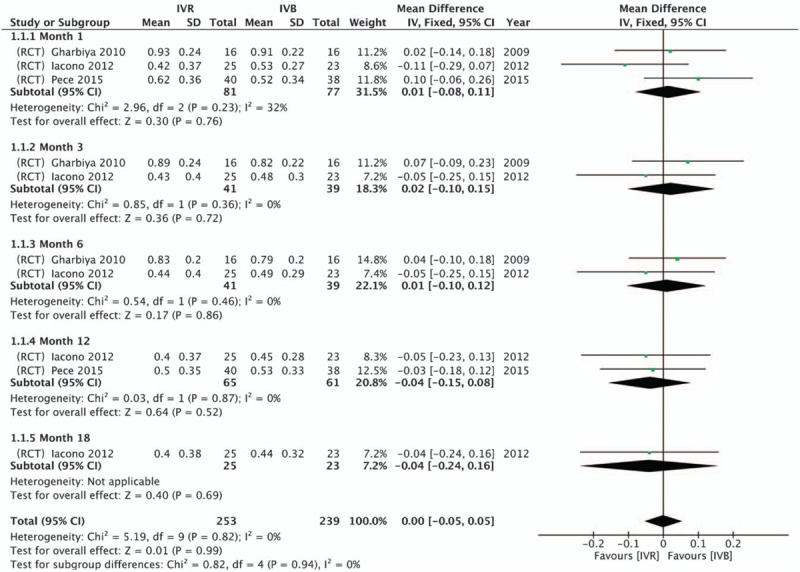
Forest plot of the mean difference in logarithm of the minimum angle of resolution BCVA using data from RCT comparisons between intravitreal IVR and IVB with a fixed- effect model.

**Figure 4:**
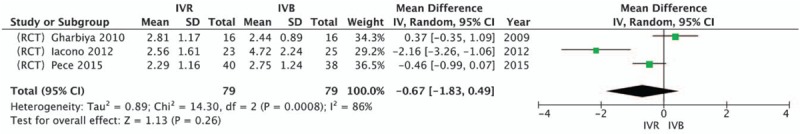
Forest plot of the mean difference in injection times using data from RCT comparisons between intravitreal IVR and IVB with a random-effect model.

Data on treatment times were extracted from the included literature and analyzed using software (Fig. 3). Results of analysis showed that the 3 articles were highly heterogeneous (*P = *.0008, *I*^2^ = 86%). Using the random effects model analysis, there was no significant difference between the number of injections in the IVR and IVB groups during the follow-up period (*Z *= 1.13, 95% CI = −1.83 to 0.49, *P = *.26 > 0.05) (Fig. 4). One of them was excluded from the analysis of heterogeneity, and it was found that the heterogeneity of each of the 2 documents was obvious. The sources of heterogeneity may be due to different study periods, and the treatment options were different from the indications of injection. The differences among the included studies would lead a large difference in the number of drug injections in each study. Limited by the number of documents included, no subgroup analysis could be performed and more high-quality RCTs that meet the inclusion criteria are required to support this.

#### 3.2.3 Central foveal thickness (CFT)

The 3 articles included described baseline levels of CFT in the 2 treatment groups, of which 2 described CFT levels at the end of follow-up. One article described CFT in the IVB group at 3, 12, and 18 months after treatment, however, only the CFT at 1 month after treatment in the IVR group was described. The incomplete data description and different follow-up time made it difficult to perform meta-analysis on the efficacy of reducing CFT between IVR and IVB groups. It is generally accepted that both IVR and IVB can significantly reduce CFT, and there is no statistical difference between the 2 therapeutic methods.

#### 3.2.4 CNV stabilization

**Figure 5:**
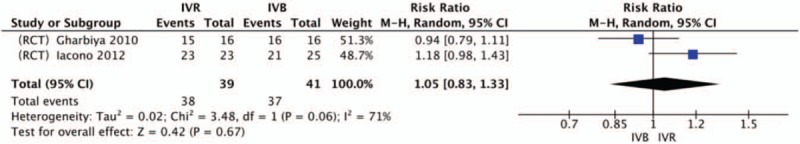
Forest plot of the CNV stabilization after 1 year using data from RCT comparisons between intravitreal IVR and IVB with a random-effect model.

Complete resolution fluorescein leakage was observed in all 16 eyes received IVB treatment and 15 out of 16 eyes received IVR treatment in Gharbiya's study. Complete CNV stabilization was observed in 21 out of 25 subjects received IVB treatment and all 23 subjects received IVR treatment in Iacono's study. A meta-analysis was performed to compare the effect of CNV stabilization between IVR and IVB (Fig. 5). No significant difference was indicated between IVR and IVB on the effect of CNV stabilization (RR: 1.05, 95% CI: 0.83 to 1.33, *P = *.67) with significant heterogeneity (*P = *.06, *I*^2^ = 71%). The heterogeneity may be due to the performance bias from doctor or technician.

## 4 Limitations

The bias and statistical difference in our results may be due to inconsistencies between the follow-up time of the included literatures and the termination event. There are few multicenter, double-blind clinical randomized controlled trials involving this direction. The small sample sizes and difficulties of obtaining complete randomized grouping information made it difficult to perform the meta-analysis. More multicenter, double-blind clinical randomized controlled studies are needed to support or correct our opinion.

## 5 Discussion

Clinical studies on anti-VEGF drugs for the treatment of choroidal neovascularization in pathological myopia began to accumulate, but most of them are uncontrolled studies, few of them are RCTs. These studies involved the comparison of the efficacy of different anti-VEGF drugs, the comparison of the efficacy of anti-VEGF drugs and photodynamic therapy (PDT), and the comparison of the efficacy of combination therapy. What kind of treatment plan should be adopted in clinical work and how dose should be mastered requires a higher level of evidence-based medical evidence.

Some non-RCTs with small sample sizes have even drawn to diametrically opposed conclusions. Previous non-RCT studies have found that the advantages of anti-VEGF drugs for PDT therapy can be withdrawn at 3 months of treatment, while RCT studies found that this advantage occurs until at 12 months of treatment. Obviously, the efficacy characteristics of different anti-VEGF drugs will be better reflected in different time periods. A longer observation period, fully randomized and consistent trial is required. However, the longest follow-up time has not exceeded 24 months currently. It can be agreed that intraocular injection of anti-VEGF drugs should be the first-line treatment of mCNV.

Ranibizumab (Lucentis) is a monoclonal antibody fragment with a molecular weight of 48 kD, which is a recombinant humanized monoclonal immunoglobulin G1 (IgG1) *κ-isotype* antibody fragment that inhibits human VEGF.^[12]^ Ranibizumab is made up of just the Fab fragment. The Fab fragment is the basis for the full-length antibody bevacizumab.^[13]^ Bevacizumab (Avastin) is a bivalent full-length monoclonal antibody with a molecular weight of 149 kD that is resistant to VEGF-A. Bevacizumab is a recombinant humanized monoclonal IgG1 antibody that inhibits human VEGF.^[14]^ Ranibizumab is approved for intravitreal injection for choroidal neovascularization and bevacizumab is approved for intravenous use for metastatic colorectal cancer. The latter is administered intravitreally off-label for choroidal neovascularization.^[13,14]^ Ranibizumab has been affinity matured to have a higher binding affinity for VEGF and conferred less antigenicity and greater retinal penetration because of the smaller molecule size.^[15]^ In the rabbit, the vitreous half-life time of 0.5 mg IVR is 2.88 days and 4.32 days for 1.25 mg IVB. No ranibizumab was detected in serum or fellow eye, while small amounts of bevacizumab were detected in serum and fellow eye.^[13,14]^ Due to the smaller molecular, ranibizumab may penetrate the retina faster and be cleared faster from the systemic circulation than bevacizumab. However, clinical trials have failed to affirm superiority for either drug. All these mechanisms bind VEGF receptors to inactivate endogenous VEGF and inhibit the migration and proliferation of vascular endothelial cells, thereby inhibiting neovascularization. The 2 drugs have similar structures and similar mechanisms. There are also studies having confirmed that there is no significant difference between ranibizumab and bevacizumab in the treatment effects of age related macular degeneration (ARMD). It may be indicated that VEGF agents play a similar role in generating CNV in either ARMD or PM.^[16]^ Only the use of bevacizumab in ophthalmology is “off-label” and the price is lower.

In summary, intraocular injection of anti-VEGF (IVR and IVB) can improve the BCVA in the treatment of choroidal neovascularization in pathological myopia. However, there is no significant difference in the BCVA improvement of the 2 therapies. There is no significant difference in the number of drug injections either, however, in previous studies, IVR required less injections. This difference probably relates to the effective concentration of the drug, the molecular weight and other factors. The comparison of IVR and IVB to reduce the foveal thickness of the retina cannot be achieved in this meta-analysis, and the results obtained in each article also tend to be insignificant.

## 6 Conclusion

Our meta-analysis indicated that both IVR and IVB could significantly increase the BCVA of patients with mCNV. The low rate of reporting ocular or systematic adverse events suggests the anti-VEGF agents may be safe. According to our meta-analysis, no significant difference was reported between the 2 treatments.

## Author contributions

**Data curation:** Haoyu Li, Yi Du.

**Formal analysis:** Qiuming Hu, Jianfeng He.

**Investigation:** Yi Du.

**Software:** Yi Du.

**Writing – original draft:** Qiuming Hu, Haoyu Li, Yi Du.

**Writing – review & editing:** Qiuming Hu, Haoyu Li, Yi Du.

Haoyu Li orcid: 0000-0002-9826-181X.
